# Genome-wide identification of growth-regulating factor transcription factor family related to leaf and stem development in alfalfa

**DOI:** 10.3389/fpls.2022.964604

**Published:** 2022-08-23

**Authors:** Yue Sun, He Li, Jiajing Wu, Kangning Zhang, Wei Tang, Lili Cong, Hongli Xie, Zeng-Yu Wang, Maofeng Chai

**Affiliations:** Key Laboratory of National Forestry and Grassland Administration on Grassland Resources and Ecology in the Yellow River Delta, College of Grassland Science, Qingdao Agricultural University, Qingdao, China

**Keywords:** gene family, growth-regulating factor, leaf size, alfalfa, expression profile

## Abstract

Growth-regulating factors (GRFs) play crucial roles in plant growth and stress response. To date, there have been no reports of the analysis and identification of the GRF transcription factor family in alfalfa. In this study, we identified 27 GRF family members from alfalfa (*Medicago sativa* L.) “Xinjiang Daye”, and analyzed their physicochemical properties. Based on phylogenetic analysis, these *MsGRFs* were divided into five subgroups, each with a similar gene structure and conserved motifs. *MsGRFs* genes are distributed on 23 chromosomes, and all contain QLQ and WRC conserved domains. The results of the collinearity analysis showed that all *MsGRFs* are involved in gene duplication, including multiple whole-genome duplication or segmental duplication and a set of tandem duplication, indicating that large-scale duplication is important for the expansion of the GRF family in alfalfa. Several hormone-related and stress-related *cis*-acting elements have been found in the promoter regions of *MsGRFs*. Some *MsGRFs* were highly expressed in young leaves and stems, and their expression decreased during development. In addition, the leaf size of different varieties was found to vary, and *MsGRF1* to *4*, *MsGRF18* to *20*, and *MsGRF22* to *23* were differentially expressed in large and small leaf alfalfa varieties, suggesting that they are critical in the regulation of leaf size. The results of this study can benefit further exploration of the regulatory functions of *MsGRFs* in growth and development, and can identify candidate genes that control leaf size development.

## Introduction

Growth-regulating factors (GRFs) are plant-specific transcription factors (TFs), that regulate plant growth and development (Kim et al., 2003; Kim and Lee, 2006; Baucher et al., 2013; Omidbakhshfard et al., 2015). The first GRF, named *OsGRF1*, was discovered 15 years ago in deep-water rice (*Oryza sativa*). *OsGRF1* was identified as the gene responding to gibberellin and differentially expressed in the internode meristems of deep-water rice (van der Knaap et al., 2000). GRFs have two conserved domains: QLQ (Gln-Q, Leu-L, Gln-Q) and WRC (Trp-W, Arg-R, Cys-C), in the N-terminal region. The QLQ domain contains sites for interaction with GRF-interacting factors (GIFs) (Kim and Kende, 2004), while the WRC domain contains a DNA-binding motif and a nuclear localization signal (Choi et al., 2004). The QLQ domains are more conservative than the WRC domains (van der Knaap et al., 2000). All eukaryotes contain QLQ; however, WRC is a plant-specific domain. In the C-terminal region of GRFs, the types and number of amino acid residues vary greatly; thus, the similarity within the family is low. Owing to the diversity of C-terminal domains, GRF proteins have functional diversity. In addition, the length of the C-terminal region determines the protein size.

Initial studies suggested that GRFs only play a role in leaf and stem development. However, recent studies have discovered that GRFs not only regulate flowering, seed, and root development, but also regulate plant longevity and participate in abiotic stress response (Hewezi et al., 2012; Kim et al., 2012; Liang et al., 2013; Debernardi et al., 2014; Liu et al., 2014). In *Arabidopsis thaliana*, *GRF*-overexpressed plants have larger leaves than wild-type plants, while the leaves of *grf* mutant plant are smaller than wild-type plant (Kim et al., 2003). *AtGRF1, 2*, and *3* control leaf size by regulating cell expansion (Kim et al., 2003), while *AtGRF1, 2, 3, 4, 5*, and *9* control leaf development through cell proliferation (Horiguchi et al., 2005; Kim and Lee, 2006; Arvidsson et al., 2011; Debernardi et al., 2014). *grf1/2/3/4* quadruple mutant plants lack shoot apical meristems (Kim and Lee, 2006). *GRF* genes are weakly expressed in mature tissues, but are highly expressed in young tissues, such as seeds, shoots, and young leaves (Liang et al., 2013). *GRF* genes encode transcription factors that bind to sequence-specific DNA, which interacts with the transcriptional cofactor GRF-INTERACTING FACTOR (GIF) to form functional transcriptional complexes that regulate cell proliferation to control leaf size (Kim et al., 2003, 2022; Kim and Kende, 2004; Horiguchi et al., 2005; Lee et al., 2009; Wang et al., 2011; Debernardi et al., 2014; Lee and Kim, 2014; Lu et al., 2020). *GRF* is negatively regulated by microRNA (miR396), and its expression is suppressed by miR396 after transcription (Wang et al., 2011; Baucher et al., 2013; Debernardi et al., 2014; Liu et al., 2014, 2021; Li et al., 2019; Szczygiel-Sommer and Gaj, 2019; Liebsch and Palatnik, 2020; Beltramino et al., 2021; Lu et al., 2021; Pegler et al., 2021; Kim et al., 2022). GRF/GIF has a universal growth-promoting effect on inflorescences and flower organs of several species. The miR396-GRF/GIF module is involved in the separation of cotyledons and flower organs (Lee et al., 2018), as well as multiple processes of flower organ growth and reproductive development (Nagai et al., 2001; Hewezi et al., 2012; Kim et al., 2012; Baucher et al., 2013). In addition, loss of GRF and GIF function usually leads to varying degrees of sterility in plants, flower organ fusion, and disturbances in the number of flower organs (Wu et al., 2014; Zan et al., 2020). Some GRFs also play an important role in abiotic stress response, including cold and salt stress (Kim et al., 2012; Khatun et al., 2017; Wallace et al., 2017; Shang et al., 2018; Li et al., 2019, 2021; Cao et al., 2020; Pegler et al., 2021). In soybean, the transcription of all *GRFs* was affected by shading. Under shade stress, almost all expressions of *GRFs* are significantly downregulated to varying degrees (Chen et al., 2019).

The identification and function of the *GRF* gene family has been studied in a variety of plants, including Arabidopsis (9) (Kim et al., 2003), soybean (22) (Chen et al., 2019), rice (12) (Choi et al., 2004), apple (16) (Zheng et al., 2018), mulberry (10) (Rukmangada et al., 2018), wheat (8) (Zan et al., 2020), and foxtail millet (Chen and Ge, 2022). However, studies on the *GRF* gene family in alfalfa are limited. As a widely used forage, alfalfa is a popular feed for livestock and poultry owing to its high yield, good forage quality, and rich nutrition. Leaves and stems, as the main harvest organ, are limiting factors for the yield and quality of alfalfa. Therefore, it is essential to study the control mechanism of leaf size during leaf development to cultivate and select germplasm resources of alfalfa with high quality.

## Results

### Identification of *MsGRFs*

In this study, we identified 27 *MsGRFs* in the alfalfa genome using hmmscan and verified them with Pfam^1^ and Conserved Domain Database (CDD) for the presence of QLQ and WRC domains. According to their chromosomal position, they were named *MsGRF1*- *MsGRF27* (Table 1). The coding sequence (CDS) length of *MsGRFs* varied slightly from 754°bp to 1,932 bp. The shortest GRF proteins were MsGRF20 and MsGRF21, which contained 251 amino acids, whereas the longest, MsGRF1, 2, and 4, had 643 amino acids. Concurrently, the physicochemical properties of *MsGRF* proteins were predicted. The theoretical molecular weight (MW) of *MsGRFs* ranged between 28,577.5 Da (MsGRF20 and MsGRF21) and 69,891.83 Da (MsGRF1, MsGRF2, and MsGRF4), and the isoelectric point (pI) ranged from 6.71 to 10.18. *MsGRF* proteins are rich in basic amino acids, with 92.59% of *MsGRF*s proteins having an isoelectric point greater than 7 (Table 1).

**TABLE 1:** Characterization of the *MsGRF* family in alfalfa.

Name	Gene ID	Chromosome	CDS (bp)	Length (aa)	MW (Da)	pI
*MsGRF1*	MS.gene24503.t1	chr1.1	1932	643	69891.83	8.16
*MsGRF2*	MS.gene00530.t1	chr1.2	1932	643	69965.86	7.33
*MsGRF3*	MS.gene051667.t1	chr1.3	1920	639	69281.96	7.33
*MsGRF4*	MS.gene66485.t1	chr1.4	1932	643	69983.90	7.33
*MsGRF5*	MS.gene025207.t1	chr2.2	1098	365	41732.92	8.61
*MsGRF6*	MS.gene64421.t1	chr2.4	1140	379	43460.96	8.61
*MsGRF7*	MS.gene06847.t1	chr3.1	1683	560	60646.77	7.81
*MsGRF8*	MS.gene057219.t1	chr3.2	1683	560	60581.66	7.81
*MsGRF9*	MS.gene06630.t1	chr3.3	1668	555	60197.41	8.24
*MsGRF10*	MS.gene013521.t1	chr3.4	1716	571	61910.21	8.24
*MsGRF11*	MS.gene53920.t1	chr4.2	963	320	35120.67	6.71
*MsGRF12*	MS.gene025012.t1	chr4.3	1014	337	37053.76	6.83
*MsGRF13*	MS.gene63415.t1	chr4.4	963	320	35120.67	6.71
*MsGRF14*	MS.gene72850.t1	chr5.1	993	330	37144.42	9.03
*MsGRF15*	MS.gene001120.t1	chr5.2	999	332	37327.66	8.97
*MsGRF16*	MS.gene028055.t1	chr5.3	999	332	37327.66	8.97
*MsGRF17*	MS.gene070101.t1	chr5.4	996	331	37246.55	8.97
*MsGRF18*	MS.gene020536.t1	chr7.2	1155	384	42583.27	8.86
*MsGRF19*	MS.gene007262.t1	chr7.4	1056	351	38775.17	9.00
*MsGRF20*	MS.gene24648.t1	chr7.4	754	251	28577.5	10.18
*MsGRF21*	MS.gene007264.t1	chr7.4	754	251	28577.5	10.18
*MsGRF22*	MS.gene007261.t1	chr7.4	1155	384	42592.28	8.86
*MsGRF23*	MS.gene051361.t1	chr7.4	813	270	30396.03	9.13
*MsGRF24*	MS.gene26508.t1	chr8.1	1110	369	42137.21	7.29
*MsGRF25*	MS.gene051816.t1	chr8.2	1110	369	42137.21	7.29
*MsGRF26*	MS.gene012219.t1	chr8.3	1110	369	42137.21	7.29
*MsGRF27*	MS.gene24830.t1	chr8.4	1110	369	42137.21	7.29

### Phylogenetic analysis of *MsGRFs*

**FIGURE 1:**
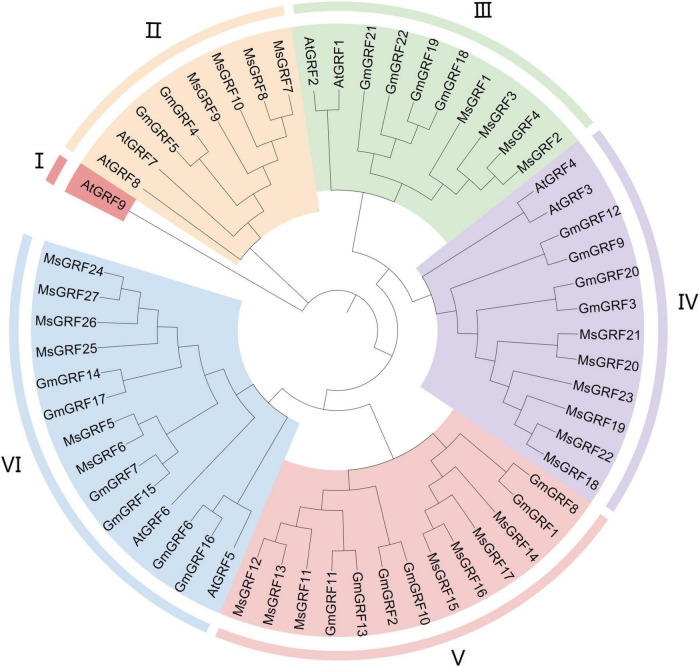
Phylogenetic analysis of growth-regulating factors (GRFs) from *Medicago sativa* L. (Ms), *Glycine max* (Gm) and *Arabidopsis thaliana* (At). MEGA 7.0 software was employed to construct a neighbor-joining phylogenetic tree with 1,000 bootstrap replications. Subgroups are highlighted with different colors.

To clearly demonstrate evolutionary relationships, we constructed a phylogenetic tree with protein sequences of 27 GRFs from alfalfa, 22 GRFs from soybean, and 9 GRFs from *Arabidopsis* using by MEGA 64 with the Neighbor-Joining (NJ) method. 58 GRFs were divided into six subgroups (I–VI) (Figure 1). Subgroup I contained only one gene, AtGRF9, and subgroups II–VI contained 8, 10, 12, 13, and 14 GRFs, respectively. Twenty-seven *MsGRF*s were assigned to subgroups II-VI: *MsGRF*s 8-10 with AtGRF7-8 and GmGRF4-5 were assigned into subgroup II; *MsGRF*s 1- 4 belonged to subgroup III with AtGRF1-2, GmGRF18-19, and GmGRF21-22; *MsGRF*s 18-23 comprised subgroup IV along with AtGRF3-4, GmGRF3, 9, 12, and 20; subgroup V only contained *MsGRF*s11-17 and GmGRF (1 to 2, 8, 10, and 13) proteins; *MsGRFs* 5, 6, and 24-27 formed subgroup VI with AtGRF 5- 6, GmGRF6, 7, and 14-17. As the functions of many Arabidopsis GRFs have been studied, the function of *MsGRF* clustered with the GRFs from soybean and Arabidopsis can be inferred from previous research. From the phylogenetic tree, it can be concluded that *MsGRFs* are more closely related to GmGRFs than to AtGRFs, which may be because both soybean and alfalfa are legumes.

### Sequence and structural analysis of *MsGRFs*

**FIGURE 2:**
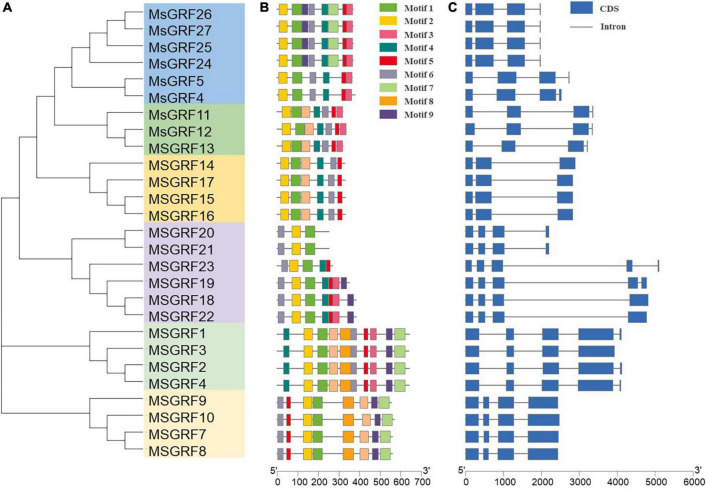
Analysis on phylogenetic relationships, motifs, and gene structure of growth-regulating factor genes from *Medicago sativa*. **(A)** Phylogenetic tree of 27 *MsGRFs* in alfalfa. **(B)** Conserved motif arrangements of *MsGRFs*. The motifs are indicated in different colored boxes with different numbers. Motifs 1 and 2 represent WRC and QLQ domains, respectively. **(C)** Exon-intron organizations of *MsGRFs*. Blue boxes indicate exons; black lines indicate introns.

Gene sequences of *MsGRFs* showed that the conserved QLQ and WRC domains existed in the N-terminal region of all *MsGRFs* (Supplementary Figure 1). To further study gene structure and evolutionary relationships, a phylogenetic tree was constructed using the protein sequences of *MsGRFs*, and their gene structure and motif characteristics were analyzed (Figures 2A–C). The homology of the *MsGRF* genes was relatively high and the motif distribution was similar, particularly in the same subgroup. Ten conserved motifs were identified using the MEME online program and renamed motifs 1–10 (Supplementary Table 1). All *MsGRFs* contained different numbers of motifs, ranging from 3 to 10. All *MsGRFs* had motif 1 and motif 2, annotated by NCBI CDD^2^ as WRC and QLQ, respectively, which are GRF-specific domains. Four members of subgroup III (*MsGRF1*, *MsGRF2*, *MsGRF3*, and *MsGRF4*) contained all ten motifs (Figures 1, 2B). Two *MsGRFs* (*MsGRF20* and *MsGRF21*) contained the least number of motifs. All *MsGRFs* contained motif 6, however, the distribution on the genes differed (Figure 2B).

Exon–intron structures clearly showed that the *MsGRFs* contained two to four introns (Figure 2C). In all, 18 of the 27 *MsGRFs* contained three introns; five *MsGRFs* (*MsGRF1*, 2, 4, 19, and 23) contained four introns; four genes (*MsGRF14*, 15, 16, and 17) contained two introns. Genes that were closely related in the phylogenetic tree had approximately the same distribution area of exons and introns. The members of each subgroup in the phylogenetic tree were similar in size and contained similar genetic structures (Figures 1, 2C). All the members of each subgroup contained the same number and similar length of exons. The length of each *MsGRF* differed depending on the length of the intron. The length of the CDS of *MsGRF23* was only 813°bp, while its full length genomic DNA was the longest. In general, motif distribution and gene structure indicate the evolutionary relationship between *MsGRFs*.

### Gene duplication and collinearity analysis

**FIGURE 3:**
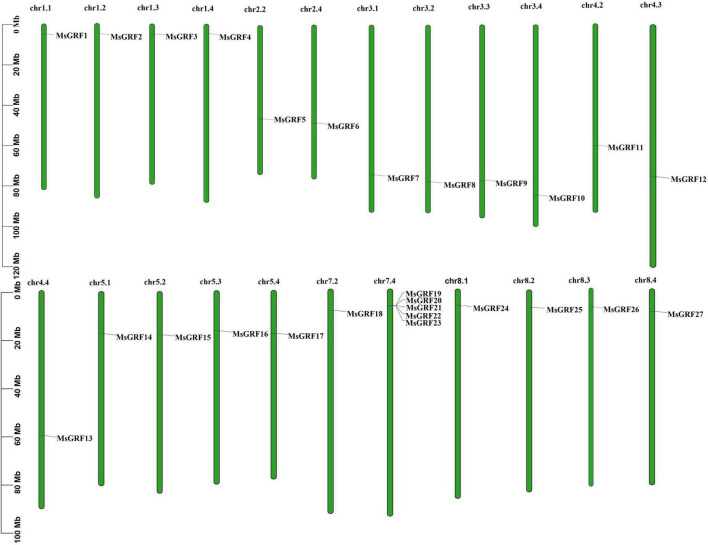
The distribution of *MsGRFs* on alfalfa chromosomes. The green bars represent each chromosome, and the black lines label the position of each *MsGRF* gene.

All *MsGRFs* were unevenly distributed on 23 chromosomes of alfalfa (Figure 3), and were not identified on the nine other chromosomes of alfalfa (2n = 4x = 32). Chromosome 7.4 (chr7.4) contains five *MsGRF* genes. Only one *MsGRF* gene was found on chr1.1, chr1.2, chr1.3, chr1.4, chr2.2, chr2.4, chr3.1, chr3.2, chr3.3, chr3.4, chr4.2, chr4.3, chr4.4, chr5.1, chr5.2, chr5.3, chr5.4, chr7.2, chr8.1, chr8.2, chr8.3, and chr8.4. For chromosomes 1, 3, 5, and 8, each allele chromosome had one *MsGRF* gene, whereas on chromosomes 2 and 7, only allele chromosomes x.2 and x.4 had *MsGRF* genes. On chromosome 4, only allele chromosome 4.1 had no *MsGRF* gene. On chromosome 6, the *MsGRF* gene has not yet been identified in each chromosome allele.

**FIGURE 4:**
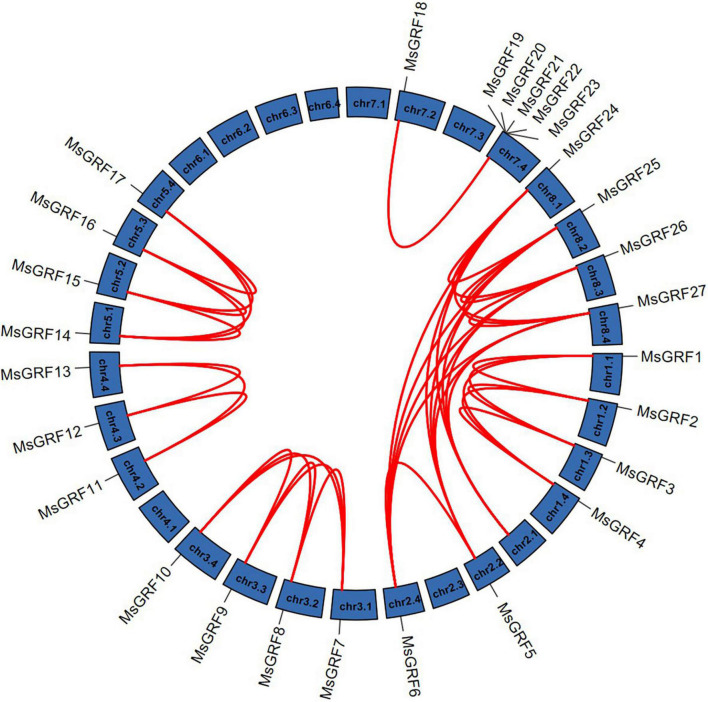
Synteny analysis of *MsGRFs* genes in alfalfa. Red lines indicate the replicated *MsGRFs* gene pairs in alfalfa.

Gene duplication is considered as one of the primary driving forces in the evolution of genomes and genetic systems. To study the gene duplication relationship of the alfalfa *GRF* family, collinearity analysis of *MsGRFs* was performed using Tbtools (Figure 4). All *MsGRFs* are involved in the duplication process, including tandem duplication, whole-genome duplication (WGD) or segment duplication. *MsGRF19*-*MsGRF23* is a set of tandem duplications located on chr7.4. Other duplicated gene pairs are genome-wide duplication or segment duplications. The non-synonymous substitution rates (Ka) and synonymous substitution rates (Ks) for each duplicated gene pair were calculated (Supplementary Table 2). The Ka/Ks values of all gene pairs were less than 1, indicating that the *MsGRF* gene family is subject to purifying selection.

### *Cis*-acting elements analysis of *MsGRFs*

**FIGURE 5:**
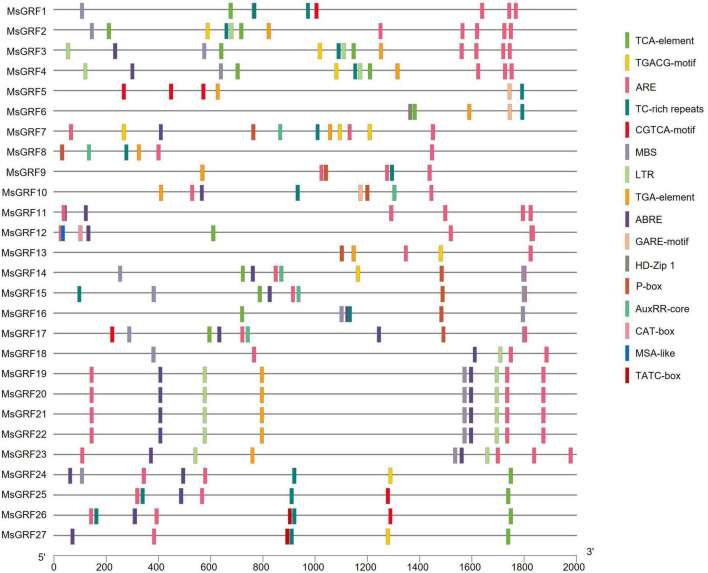
The *cis*-acting element contained in the 2 kb promoter sequence of the *MsGRF* gene. Different *cis*-elements are indicated by different colored rectangles and placed in the matching position on the promoter.

The online *cis*-element database PlantCARE was used to analyze the promoter sequences (upstream 2,000 bp) of *MsGRFs*. Conserved core elements TATA-box and enhancement elements CAAT-box in the promoter sequences were observed, which conformed to the basic structural characteristics of eukaryotic gene promoters. The promoter sequence also contained many elements related to hormonal and abiotic stress responses (Figure 5). Hormone-responsive elements include jasmonic acid-responsive elements (CGTCA-motif and TGACG-motif), salicylic acid *cis*-acting element (TCA-element), gibberellin-responsive elements (GARE motif, P-box and TATC-box), abscisic acid-responsive elements (ABRE), and auxin-responsive elements (AuxRR-core, TGA-element). Abiotic stress response elements include the anaerobic inducible element (ARE), disease resistance and stress response element (TC-rich repeats), low temperature responsive *cis*-acting element (LTR), and MYB binding site involved in drought-inducibility (MBS). In addition, we found certain unique *cis*-acting elements in the promoter sequence: CAT-box (*cis*-acting regulatory element related to meristem expression), MSA-like (*cis*-acting element involved in cell cycle regulation), and HD-Zip 1 (element involved in differentiation of the palisade mesophyll cells). Each *MsGRF* contains at least one hormone-related *cis*-element and one stress-related *cis*-acting element, however, the types vary.

### Expression analysis of *MsGRFs* in different developmental stages

**FIGURE 6:**
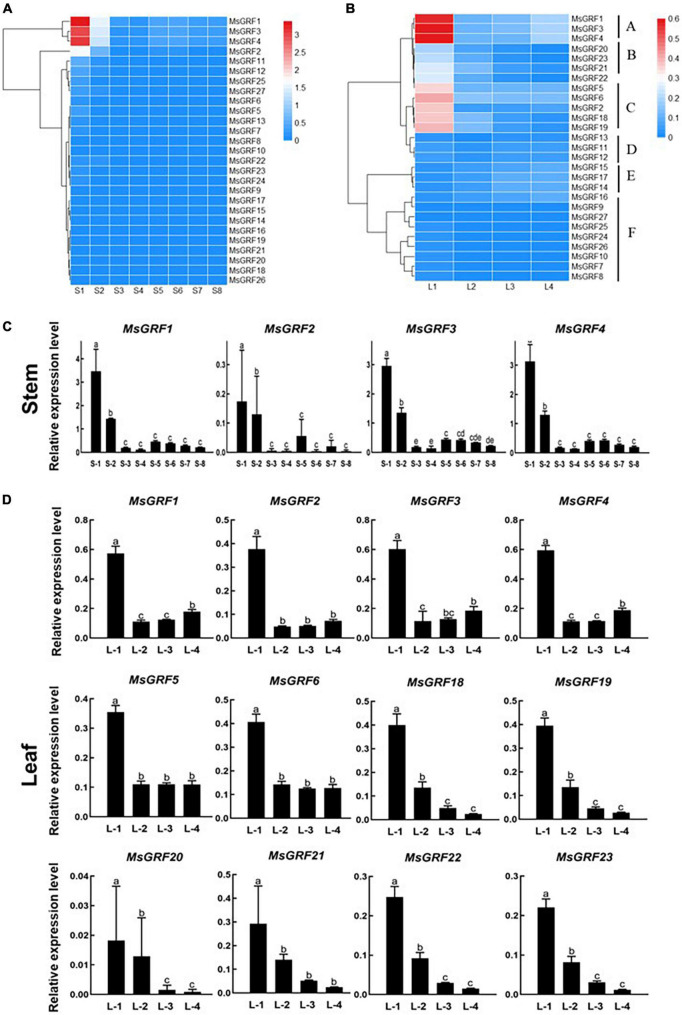
Expression profiling of *MsGRFs.* **(A)** Expression profiles of *MsGRFs* at different developmental stages of stem internodes. Each stem internode starting from the apex is used as a developmental stage, labeled as Stem-1 to 8. **(B)** Expression profiles of *MsGRFs* at different developmental stages of leaves clustered into A to F six clades. L1 to L4 indicates the leaf development according to leaf position, and the first leaf that has not fully unfolded is regarded as the first stage of leaf development (L1). **(C,D)** qRT-PCR quantification of gene expression levels of selected *MsGRF* genes from **(A,B)** displayed in a column chart. The different letters (a, b, c, etc.) indicate the significant difference at *P* < 0.05 by Student’s *t*-test analysis.

To analyze the expression of *MsGRFs* at different developmental stages of stems and leaves, the expression levels of 27 genes at different growth and developmental stages were verified by qRT-PCR, and the results were visualized as heatmaps (Figures 6A,B and Supplementary Table 3). Each stem internode, from the apex to base of the stem, is used as a developmental stage, labeled as S1 to S8. The first leaf that has not fully unfolded is regarded as the first stage of leaf development (L1), and is then divided into L1 to S4 according to leaf position (Supplementary Figure 2).

The expression of *MsGRFs* in stems is shown in the Figure 6A. According to the expression patterns at different developmental stages of the stem, most of the *GRF* family genes were weakly expressed. Compared with other *MsGRF* genes, the expression levels of *MsGRF1 to 4* were significantly higher. These four genes were most strongly expressed in the S1 stage, followed by the S2 stage, and weakly expressed in the stems at other developmental stages. The qRT-PCR results in column chart showed clearly that the expression levels of these four *MsGRFs* decreased dramatically from the first period, increased a little in the S5 period, and then decreased gradually (Figure 6C). Overall, the expression of the four *MsGRFs* gradually decreased during stem growth and development. In conclusion, it is speculated that *MsGRFs1-4* plays an important role in regulating stem development.

In the expression profile of leaves (Figure 6B), *MsGRFs* can be clustered into six clades, denoted by A-F. The *MsGRF* genes of cluster F were negligibly expressed. Cluster D and E expression was weak, but the expression level of cluster E increased slightly at the L3 and L4 stage. In contrast, cluster A was strongly expressed in leaves, especially at the L1 stage. The expression levels of clusters B and C were higher in the early stages of leaf development, and differed significantly from those in the other stages (Figure 6B). The expression of *MsGRFs1-6* was the strongest in the L1 stage, fluctuated in the L2, L3, and L4 stages, and showed a downward trend in general, revealed by qRT-PCR analysis in column chart (Figure 6D). The expression of *MsGRFs18* to *23* reached the summit at the L1 stage, and then decreased significantly during leaf development (Figure 6D). In summary, the expression of *MsGRFs* was high in the early stages of leaf development and weak in mature leaves. These results indicate that *MsGRF1-6* and *MsGRF18-23* play important roles in the leaf development.

### Identification of large and small leaf alfalfa varieties and growth-regulating factor gene expression analysis

**FIGURE 7:**
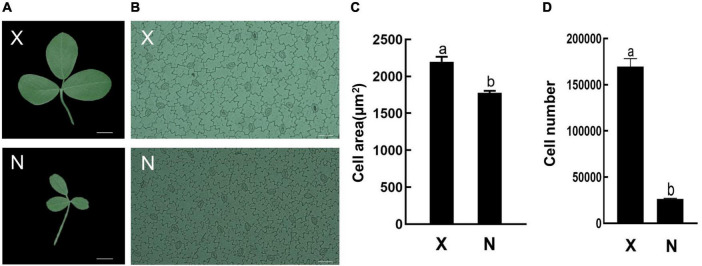
Leaf morphology and lower epidermal cells observed under microscope. **(A)** Morphological observation of large (X, “Xinjiang Daye”) and small (N, “Nei 1 × Nei 2”) leaves. Scale bar, 1 cm. **(B)** Epidermal cells of large (X, “Xinjiang Daye”) and small (N, “Nei 1 × Nei 2”) leaves under microscope. Scale bar, 50 μm. **(C)** The average area of a single epidermal cell in the leaf of “Xinjiang Daye” and “Nei 1 × Nei 2”. **(D)** Estimates of the average total cells number in a single leaf of “Xinjiang Daye” and “Nei 1 × Nei 2”. The letters (a, b) indicate the significant difference at *P* < 0.05 by Student’s *t*-test. The L4 stage leaves were used for the observation.

**FIGURE 8:**
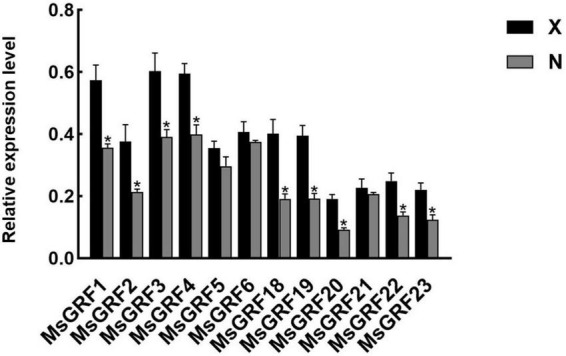
Quantification of the expression levels of selected *MsGRFs* in the leaves of X, “Xinjiang Daye” and N, “Nei 1 × Nei 2” using qRT-PCR. Vertical bars indicate standard deviation. The asterisk (*) indicates the significant difference at *P* < 0.05 by Student’s *t*-test.

To analyze GRF function on leaf development, the small leaf, and large leaf alfalfa varieties was investigated. The leaves of “Xinjiang Daye” were larger than those of the “Nei 1 × Nei 2” varieties (Figure 7A). To clarify whether the development of alfalfa leaf size was regulated by cell proliferation or expansion, the number of lower epidermal cells of the L4 stage leaves from the two varieties under a microscope and the average cell area of a single epidermal cell was investigated (Figures 7A,B). The average area of a single lower epidermal cell in “Xinjiang Daye” is larger than “Nei 1 × Nei 2” (Figure 7C). The average cell number of a single leaf in “Xinjiang Daye” is much higher than that in “Nei 1 × Nei 2” (Figure 7D). These results implied that leaf size is regulated by both cell proliferation and expansion. According to the expression of *MsGRFs* at different developmental stages of leaves (Figure 6D), we selected *MsGRFs* with high expression at L1 stage for qRT-PCR analysis to verify their expression in large leaves (“Xinjiang Daye”) and small leaves (“Nei 1 × Nei 2”) alfalfa varieties. The selected *MsGRF*s were highly expressed in these two varieties, but they had differences in the expression levels. It was found that the expression levels of *MsGRF1* to *4, MsGRF18* to *20*, and *MsGRF22* to *23* were much higher in “Xinjiang Daye” than that in “Nei 1 × Nei 2” (Figure 8).

## Discussion

The GRF family, as a class of plant-specific transcription factors, plays an important role in plant growth and development (Kim et al., 2003; Kim and Lee, 2006; Baucher et al., 2013; Omidbakhshfard et al., 2015). The GRF gene family has been identified and studied in many species, but has not been reported in alfalfa. Existing studies have confirmed that the GRF family can regulate the development of roots, stems, leaves, flowers, and fruits, the maintenance of shoot apical meristems, regulate plant longevity and respond to abiotic stresses (van der Knaap et al., 2000; Kim et al., 2003; Kim and Kende, 2004; Horiguchi et al., 2005; Kim and Lee, 2006; Marcotrigiano, 2010; Wang et al., 2011; Hewezi et al., 2012; Baucher et al., 2013; Debernardi et al., 2014; Lee and Kim, 2014; Wu et al., 2014; Tao et al., 2016; Beltramino et al., 2018; Lee et al., 2018; Lockhart, 2018; Zhang D. et al., 2018; Lu et al., 2020; Kim et al., 2022). In Arabidopsis, GRF genes regulate leaf size *via* cell proliferation and expansion (Kim et al., 2003; Kim and Kende, 2004; Horiguchi et al., 2005; Wang et al., 2011; Debernardi et al., 2014; Beltramino et al., 2018; Shimano et al., 2018). The leaves are the main site of photosynthesis (Tsukaya, 2014), and the main harvesting organ in alfalfa. Therefore, it is essential to study the regulatory mechanisms of the GRF gene family in the development of alfalfa leaf size. In this study, we performed bioinformatic analysis of 27 GRF gene family members identified in alfalfa variety “Xinjiang Daye”, and predicted the physicochemical properties and *cis*-acting elements of *MsGRFs*. To clarify the evolutionary relationship of the GRF gene family, a phylogenetic tree was constructed and its gene structure and motif distribution were studied. The replication relationship between genes was analyzed using chromosomal location and collinearity analysis. The expression patterns of *MsGRFs* at different growth and developmental stages of the leaves and stems were quantitatively analyzed by qRT-PCR.

The GRF family contains QLQ and WRC conserved domains at the N-terminus, and the WRC region contains DNA-binding motifs and nuclear localization signal regions, which can combine with the *cis*-acting regions of downstream genes to regulate the expression of such genes (Kim and Kende, 2004). The QLQ domain can combine with GIF to form a transcriptional activator and play a regulatory role (Choi et al., 2004). These 27 genes were verified to contain QLQ and WRC domains (Supplementary Figure 1); therefore, they were finally identified as GRF family members. The protein lengths of *MsGRFs* ranged from 251 to 643 amino acids. Subsequently, the physicochemical properties of the *MsGRFs* were predicted, including isoelectric point and molecular weight. The theoretical MW of *MsGRFs* was between 28,577.5 Da and 69,891.83 Da, and the isoelectric point (pI) was between 6.71 and 10.18 (Table 1).

Phylogenetic analysis showed that 27 *MsGRFs* were divided into six subgroups according to their phylogenetic relationships (Figure 1). *AtGRF1, 2, 3* have been shown to regulate leaf size through cell proliferation and expansion (Kim et al., 2003; Kim and Kende, 2004; Horiguchi et al., 2005; Hewezi et al., 2012; Beltramino et al., 2018), therefore, the function of *MsGRFs* in the same subgroup can be inferred according to clustering in the evolutionary tree, which provides a foundation for future studies on the mechanism of GRFs that control leaf size development. The gene structure and motif distribution were consistent with the phylogenetic results, which confirmed the phylogenetic relationship among *MsGRF* genes (Figure 2). Members of the same subgroup contain similar gene structures and conserved motifs. Studies have shown that most genes in the *GRF* family contain three introns (Wang et al., 2014; Shang et al., 2018; Zhang J. et al., 2018), which is consistent with our results of gene structure analysis (Figure 2C).

Gene duplication is considered to be one of the primary driving forces in the evolution of genomes and genetic systems. Tandem duplication events and large-segment duplication events are considered the main reasons for the expansion of gene families in the genome (Levasseur and Pontarotti, 2011). *MsGRFs* were distributed on 23 chromosomes, and no *GRF* family members were identified in any of the copies of chr 6.1–6.4 (Figure 3). According to sequence alignment, the sequences of the *MsGRFs* on each chromosomal copy were highly homologous. The results of the collinearity analysis showed that the *MsGRF* gene family was expanded by large segment duplication. The *MsGRFs* in alfalfa were involved in gene duplication events (Figure 4). Chromosome 7.4 contains five genes identified as tandem repeats of *MsGRFs*, which are arranged in neighboring positions, and form a gene cluster with similar sequences. In other species, such as soybean, wheat, and foxtail millet, duplication events of the GRF gene family have also been demonstrated, (Chen et al., 2019; Zan et al., 2020; Chen and Ge, 2022). The Ka/Ks of all replicating gene pairs was less than 1 (Supplementary Table 2), and most of the non-synonymous substitutions were harmful, indicating that the environmental selection pressure during the evolution process was negative, and the *MsGRF* genes were selected for purification.

*Cis*-acting elements are DNA sequences that exist upstream of a gene and participate in the regulation its expression. They do not encode any protein but only provide a binding site for action (Hernandez-Garcia and Finer, 2014). In this study, we predicted that *cis*-acting elements located 2,000 bp upstream of the promoter using PlantCARE (Figure 5). The promoter sequences of *MsGRFs* contain hormone-related *cis*-acting elements and stress-related *cis*-acting elements, among which ARE is the most widely distributed, followed by ABRE. In addition, only *MsGRF6* contained HD-Zip 1 (an element involved in differentiation of the palisade mesophyll cells), and *MsGRF12* contained CAT-box (*cis*-acting regulatory element related to meristem expression) and MSA-like (*cis*-acting element involved in cell cycle regulation) element. Each *MsGRF* contained abiotic stress-related *cis*-acting elements, indicating that these genes responded to different stresses. Based on these results, candidate genes are provided for studies related to abiotic stress.

The expression of *MsGRFs* in various tissues plays an important role in growth and development. It has been demonstrated in previous studies that the GRF gene family is strongly expressed in young tissues and weakly expressed in mature tissues (Zhang et al., 2008; Khatun et al., 2017; Zheng et al., 2018; Zhou et al., 2018; Zan et al., 2020; Tang et al., 2021). In this study, the expression patterns of the *MsGRF* family were similar in leaves and stems, with high expression in young stems and leaves, which decreased with growth and development (Figure 7). *MsGRFs1-4* were significantly expressed in leaves and stems, indicating that these genes play an important role in regulating their growth and development. By observing the lower epidermal cells of different sizes of leaves from different varieties, we found that the size of alfalfa leaves was controlled by cell proliferation and expansion (Figure 7). The expression of several *MsGRF* genes was significantly different in large and small leaf alfalfa varieties, such as *MsGRF1* to *4*, *MsGRF18* to *20*, and *MsGRF22* to *23* (Figure 8), which may be related to the regulation of leaf size. As the main site of photosynthesis, the leaves are also the main harvesting organs of alfalfa. Studying the control mechanism of leaf size is crucial to understanding the ecology and increasing production of alfalfa (Liu et al., 2012, 2021). According to the expression of *MsGRF* genes in different developing leaves, many candidate genes have been identified, and the key genes controlling leaf size need to be further investigated.

## Conclusion

In this study, 27 GRF family members in alfalfa were identified and their basic characteristics and functions were subjected to preliminarily analysis. QLQ and WRC are two domains unique to the GRF gene family that helped us identify *MsGRFs* in alfalfa. To study the evolutionary relationships between GRFs, a phylogenetic tree was constructed and divided into six subgroups. Members of the same subgroup have similar gene structures and motif distributions. In alfalfa, there are 23 chromosomes with GRF family genes, among which chr7.4 contains five genes and the other chromosomes only contain one gene. All *MsGRFs* are involved in gene duplication events including tandem duplication, whole-genome duplication, and segment duplication. The results of the collinear analysis showed that gene duplication facilitated the expansion of *MsGRFs*. The upstream regions of the promoters of *MsGRFs* all contain one or more hormone or stress-related *cis*-acting elements. *MsGRF1-4* were strongly expressed in young stems and leaves, whereas *MsGRF5, 6* and *MsGRF18-23* were only highly expressed in young leaves and not in stem. The expression of *MsGRF1-4, MsGRF18*-*20*, and *MsGRF22*-*23* were significantly different in large and small leaf alfalfa varieties. In conclusion, these results lay a foundation for us to further study the function and regulatory mechanism of the alfalfa *GRF* gene family in leaf development and screen the key genes for controlling leaf size.

## Materials and methods

### Identification of *MsGRFs*

The “Xinjiang Daye” genome used in this article is publicly available in the NCBI database under project PRJNA540215, and the genome assembly files are available at https://figshare.com/projects/whole_genome_sequencing_and_assembly_of_Medicago_sativa/66380 (Chen et al., 2020). Alfalfa protein sequences and genome annotations were downloaded from the Alfalfa Breeder’s Toolbox.^3^ Transcription factor prediction and blast analysis were performed using Majorbio Cloud Platform.^4^ Analysis of transcription factors was performed using hmmscan with parameter *E*-value ≤ 1e^–5^ (Lozano et al., 2015). Finally, sequences were verified with Pfam(see text footnote 1) and CDD.^5^ The genes containing the WRC and QLQ domains were considered to be *MsGRFs*. The ExPASy proteomics server^6^ was used to predict the physicochemical properties of each *MsGRF* protein, including the molecular weight (MW) and theoretical isoelectric point (pI) (Wilkins et al., 1999).

### Sequence and phylogenetic analysis

Full-length amino acid sequences of GRF in alfalfa were aligned using MEGA 7.0 (Kumar et al., 2016). Conserved motifs for predicted *MsGRFs* protein sequences were identified using the MEME online program^7^ with default settings, except that the motif number was set as 10 (Bailey et al., 2009). Gene structure and motif distribution were visualized using the TBtools software (Chen et al., 2018). Nine Arabidopsis GRF sequences from The Arabidopsis Information Resource (TAIR)^8^ and 22 GRF sequences of soybean from Phytozome v13^9^ were used to construct a phylogenetic tree (Supplementary Table 5). MEGA 7.0 was used to construct phylogenetic trees using the neighbor-joining method with Poisson model, pairwise deletion, and 1,000 bootstrap replications (Kumar et al., 2016). The *cis*-acting elements in the 2,000 bp upstream sequences of the promoter of *MsGRFs* were predicted using PlantCARE^10^ (Lescot et al., 2002), and TBtools was used to visualize the *cis*-acting elements.

### Chromosome distribution, gene duplication, and collinearity analysis

The chromosomal location of the alfalfa *GRF* gene was obtained from the genome assembly files,^11^ and the chromosomal distribution was mapped using TBtools. Collinearity analysis of 27 *MsGRFs* gene was performed using TBtools software to detect gene duplication events. Based on the results of the collinearity analysis, calculation of non-synonymous (Ka) and synonymous (Ks) substitutions for each pair of duplicated genes were made using TBtools. The ratio of Ka/Ks was used to do the analysis of selection pressure.

### Quantitative real-time polymerase chain reaction analysis

To investigate the expression patterns of *MsGRFs*, total RNA from different tissues were used for qRT-PCR (Schmittgen and Livak, 2008). Total RNA was extracted using the Takara MiniBEST Plant RNA Extraction Kit (Takara Bio Inc., Kusatsu, Japan), and the RNAs were reverse transcribed into cDNAs using HiScript III^®^ RT SuperMix for qRT-PCR (+ gDNA wiper) (Vazyme Biotech Co., Ltd., Nanjing, China). ChamQ SYBR Color qRT-PCR MasterMix (Vazyme Biotech Co., Ltd., Nanjing, China) was used for qRT-PCR, and the MsUBC Q-2F gene was used as a reference gene, each of which had three technical replicates. Beacon Designer 7.9 was used to design real-time quantitative primers, and the sequences of the primers used for qRT-PCR was listed in Supplementary Table 4.

### Plant material and growth condition

In this experiment, we used cultivated “Xinjiang Daye” and “Nei 1 × Nei 2” alfalfa as plant material. The alfalfa seeds were placed in a petri dish containing H_2_O and then placed in a germination bag. After seven days, the germinated seedlings were transferred to 1/2 Hoagland’s nutrient solution for growth and cultivation, during which the nutrient solution was changed every 3 days. Plants were placed in an artificial climate incubator with a 16-hour photoperiod, day and night temperature of 25°C/22°C, and relative humidity of 60–70%. The plant materials used for the morphological observation were “Nei 1 × Nei 2” and “Xinjiang Daye” alfalfa cultivated in the same environment.

To analyze the expression patterns in different growth stages, the leaves and stems of 30-day-old alfalfa were selected. The leaves were divided into four developmental stages (L1, L2, L3, and L4), and the stems of the same plant were divided into eight developmental stages (S1 to S8) according to the order of stem nodes from the apex to base (Supplementary Figure 2). All samples were immediately frozen in liquid nitrogen and stored at −80° until use.

## Data availability statement

The datasets presented in this study can be found in online repositories. The names of the repository/repositories and accession number(s) can be found in the article/Supplementary material

## Author contributions

MC, YS, and HL designed and planned the experiments. HL, YS, JW, and WT performed the experiments. YS and HL analyzed the data and wrote the manuscript. MC, LC, HX, and Z-YW revised the manuscript. All authors read, revised, and approved the final manuscript.

## Footnotes

1
http://pfam.xfam.org

2
https://www.ncbi.nlm.nih.gov/Structure/cdd/wrpsb.cgi

3
https://www.alfalfatoolbox.org/

4
www.majorbio.com

5
https://www.ncbi.nlm.nih.gov/cdd

6
https://web.expasy.org/protparam/

7
http://meme-suite.org/tools/meme

8
http://www.arabidopsis.org

9
https://phytozome-next.jgi.doe.gov

10
http://bioinformatics.psb.ugent.be/webtools/plantcare/html/

11
https://figshare.com/projects/whole_genome_sequencing_and _assembly_of_Medicago_sativa/66380

## Supplementary material

Supplementary Figure 1 Protein sequence alignment of 27 *MsGRFs* and conserved domains of QLQ and WRC.

Supplementary Figure 2 Different developmental stages of stems and leaves in alfalfa. L1 represents the first leaf that is not fully expanded, then L2, L3, and L4 are defined according to leaf position. From top to bottom in the stem, each stem node is used as a developmental stage, represented by S1–8.
