# Supplementary figures and images for: Genome-wide identification of growth-regulating factor transcription factor family related to leaf and stem development in alfalfa
Source: Front Plant Sci. 2022 Aug 23;13:964604. doi: 10.3389/fpls.2022.964604 (PMC9445573; doi:10.3389/fpls.2022.964604)

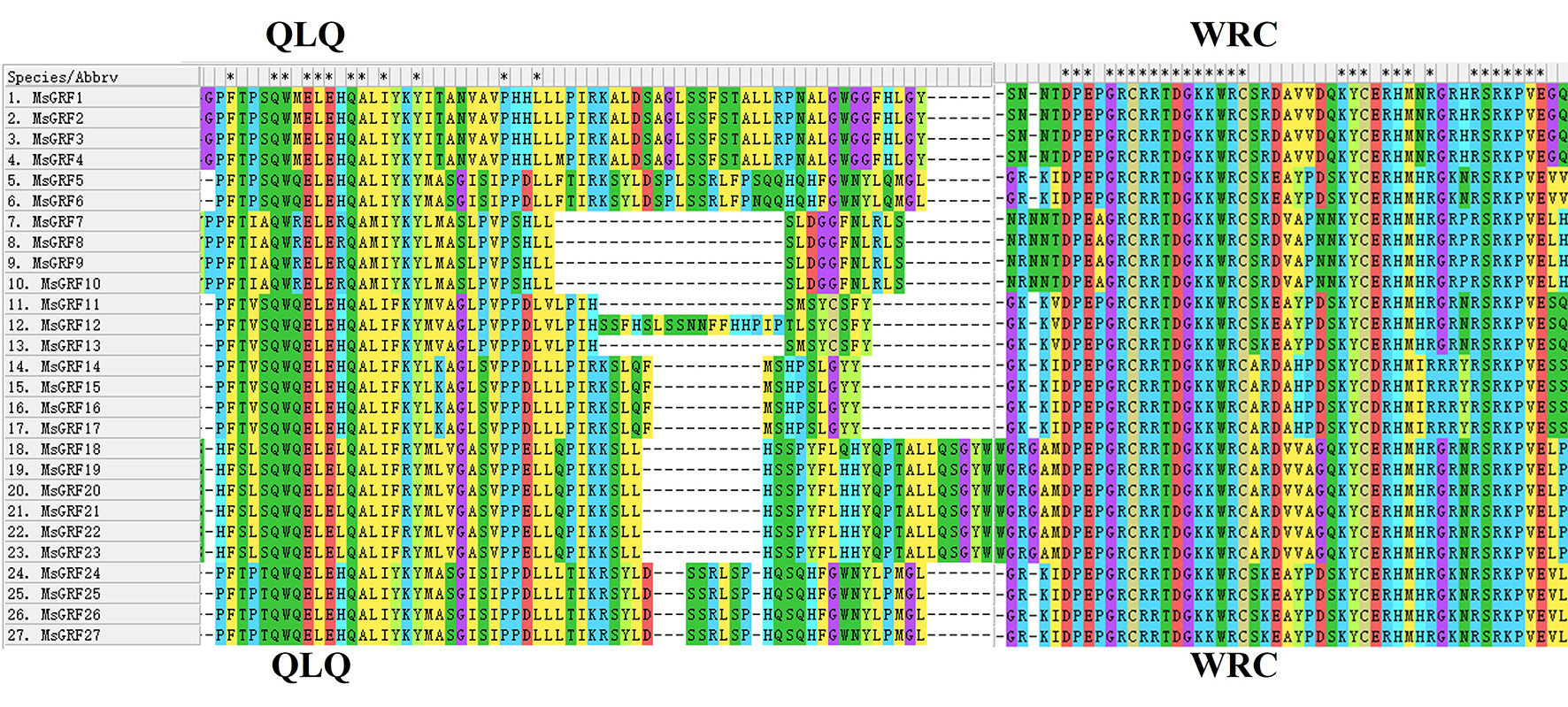

Supplement: Supplementary Figure 1 — Protein sequence alignment of 27 MsGRFs and conserved domains of QLQ and WRC. [file Image_1.JPEG]

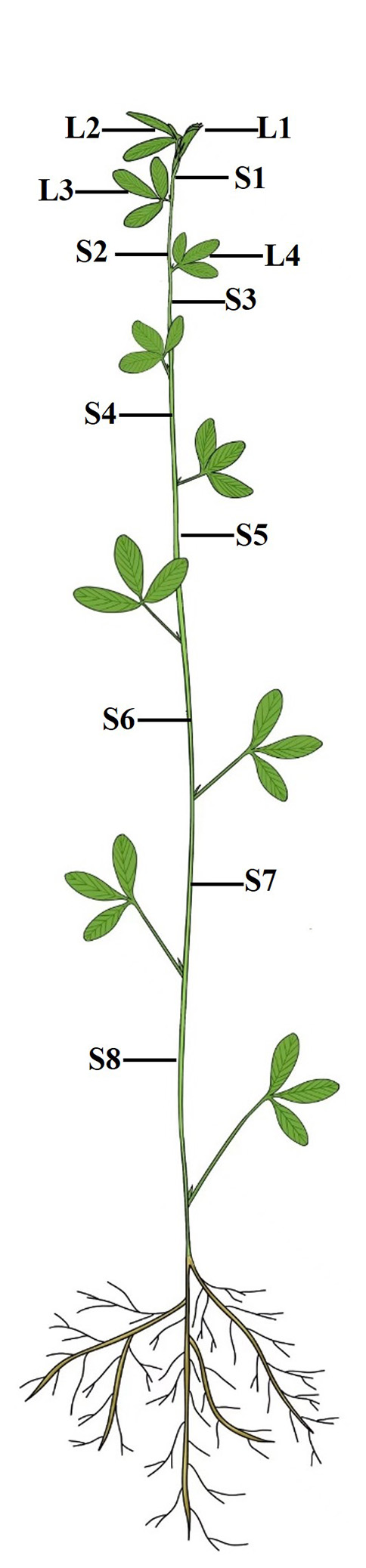

Supplement: Supplementary Figure 2 — Different developmental stages of stems and leaves in alfalfa. L1 represents the first leaf that is not fully expanded, then L2, L3, and L4 are defined according to leaf position. From top to bottom in the stem, each stem node is used as a developmental stage, represented by S1–8. [file Image_2.JPEG]
